# Digestive Gland from *Aplysia depilans* Gmelin: Leads for Inflammation Treatment

**DOI:** 10.3390/molecules200915766

**Published:** 2015-08-28

**Authors:** Andreia P. Oliveira, Alexandre Lobo-da-Cunha, Marcos Taveira, Marta Ferreira, Patrícia Valentão, Paula B. Andrade

**Affiliations:** 1 REQUIMTE/LAQV, Laboratório de Farmacognosia, Departamento de Química, Faculdade de Farmácia, Universidade do Porto, Rua de Jorge Viterbo Ferreira, No. 228, 4050-313 Porto, Portugal; E-Mails: andreiapsoliveira@gmail.com (A.P.O.); marcos.taveira@gmail.com (M.T.); martasbcf@gmail.com (M.F.); valentao@ff.up.pt (P.V.); 2 Department of Microscopy, Institute of Biomedical Sciences Abel Salazar (ICBAS), University of Porto, Rua de Jorge Viterbo Ferreira, No. 228, 4050-313 Porto, Portugal; E-Mail: alcunha@icbas.up.pt; 3 Interdisciplinary Centre of Marine and Environmental Research (CIIMAR), Rua dos Bragas 289, 4050-123 Porto, Portugal

**Keywords:** *Aplysia depilans* digestive gland, fatty acids, carotenoids, anti-inflammatory potential

## Abstract

The exploitation of marine organisms for human nutritional and pharmaceutical purposes has revealed important chemical prototypes for the discovery of new drugs, stimulating compounds isolation and syntheses of new related compounds with biomedical application. Nowadays, it is well known that inflammatory processes are involved in many diseases and the interest in the search for marine natural products with anti-inflammatory potential has been increasing. The genus *Aplysia* belongs to the class *Gastropoda*, having a wide geographical distribution and including several species, commonly known as sea hares. *Aplysia depilans* Gmelin is usually found in the Mediterranean Sea and in the Atlantic Ocean, from West Africa to the French coast. In these marine organisms, most of the digestion and nutrient absorption occurs in the digestive gland. This work aimed to explore the chemical composition and bioactivity of the methanol extract from *A. depilans* digestive gland. Therefore, fatty acids and carotenoids were determined by GC-MS and HPLC-DAD, respectively. Twenty-two fatty acids and eight carotenoids were identified for the first time in this species. The *A. depilans* digestive gland revealed to be essentially composed by polyunsaturated fatty acids (PUFA) and xanthophylls. Regarding the anti-inflammatory potential in RAW 264.7 cells stimulated with lipopolysaccharide, it was observed that this matrix has capacity to reduce nitric oxide (NO) and L-citrulline levels, which suggests that its compounds may act by interference with inducible nitric oxide synthase. Taking into account the results obtained, *A. depilans* digestive gland may be a good source of nutraceuticals, due to their richness in health beneficial nutrients, such as carotenoids and long-chain PUFA.

## 1. Introduction

**Figure 1:**
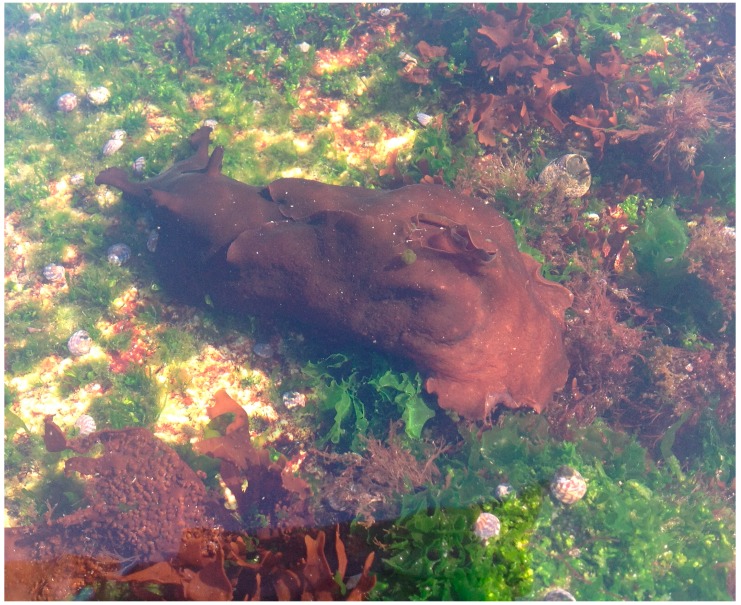
*Aplysia depilans* Gmellin.

The marine environment comprises an immense diversity of living organisms, which is still underexplored compared to terrestrial ones. Marine organisms have been shown to be an excellent source of bioactive compounds that can be applied in several areas, namely in agricultural, food, textile and pharmaceutical industries. In the last few decades, a large number of metabolites with diverse biological capacities has been intensively isolated from marine organisms, such as algae, sponges and mollusks [1]. The genus *Aplysia*, belongs to the class *Gastropoda*, possesses a wide geographical distribution and includes several species, commonly known as sea hares. These mollusks are soft-bodied marine invertebrates, without physical protection, and ingest relatively large fragments of algae, requiring a complex digestive system to extract nutrients from the food they consume. *Aplysia depilans* (Gmelin, 1791) (Figure 1) is usually found in the Mediterranean Sea and in the Atlantic Ocean, from West Africa to the French coast [2,3]. In these marine organisms, most of the digestion and nutrient absorption occurs in the digestive gland; the waste coming from it returns to the stomach, being conducted into the incurrent channel of the caecum. Subsequently, the undigested substances are carried by ciliary currents and muscular activity, being transferred to the excurrent channel of the caecum, where the fecal rods are formed [4,5].

Few studies have been performed with *A. depilans*: they describe the presence of two 5,8-epidioxysterols [6], the characterization of aplyolides A–E, 16- and 18-membered fatty acid lactones [7], the identification of nine brominated diterpenes and the evaluation of the *in vitro* cytotoxic activity against human tumor cell lines [8]. Regarding *A. depilans* digestive glands, studies were performed to characterize ultrastructural and cytochemical aspects [9,10]. Nevertheless, as far as we know, there is no study concerning either the metabolic composition of *A. depilans* digestive gland, or its biological potential.

Inflammation is a pathophysiological response by the organism in order to neutralize an agent that causes infection or tissue damage. The inflammatory process is characterized by the appearance of redness, heat and pain as result of a series of sequentially occurring events, such as dilatation of arterioles and venules, increased permeability of blood vessels following stasis and thrombosis, leukocyte infiltration into tissue, extravasation of blood plasma, proteolytic activity, formation of reactive oxygen and nitrogen species, necrosis, apoptosis and phagocytosis [11]. The inflammatory response is a complex process involving different signaling pathways [12]. In a general way, it is initiated by inducers (exogenous or endogenous), which activate specialized sensors to produce specific mediators [13]. Thereby, these mediators modify the functional states of tissues and organs, allowing the adaptation to the inflammatory conditions [13]. It is well known that macrophages play a critical role in the initiation, maintenance, and resolution of inflammation. They are activated and deactivated in the inflammatory process [14]. When macrophages are activated, they produce pro-inflammatory cytokines, such as interleukins 1 and 6 (IL-1 and IL-6, respectively), tumor necrosis factor α (TNF-α), and other inflammation mediators like nitric oxide (NO) and prostaglandin E2 (PGE2) [15]. Nonetheless, the overproduction of mediators is involved in many diseases, such as rheumatoid arthritis, atherosclerosis [16], asthma [17] and pulmonary fibrosis [18]. Regarding NO, it is an intracellular and intercellular signaling molecule involved in the regulation of diverse physiological and pathophysiological mechanisms in the cardiovascular, nervous and immunological systems. In fact, this short-lived free radical has an essential role in inflammation, providing an anti-inflammatory effect under normal physiological conditions [19]. Thus, the regulation of their production in tissues might be important for the treatment of inflammation.

Therefore, the aim of this study was to contribute to the knowledge of the metabolic profile of the digestive gland from *A. depilans* and of its biological properties. Fatty acids and carotenoids profiles were characterized by gas chromatography- mass spectrometry (GC-MS) and high-performance liquid chromatography with diode-array detection (HPLC-DAD), respectively, and anti-inflammatory activity was approached on lipopolysaccharide (LPS)-stimulated macrophages, by NO and L-citrulline determination. In addition, some correlations between the chemical composition and biological activity were established.

## 2. Results and Discussion

### 2.1. Metabolic Analysis

#### 2.1.1. Fatty Acids

**Figure 2:**
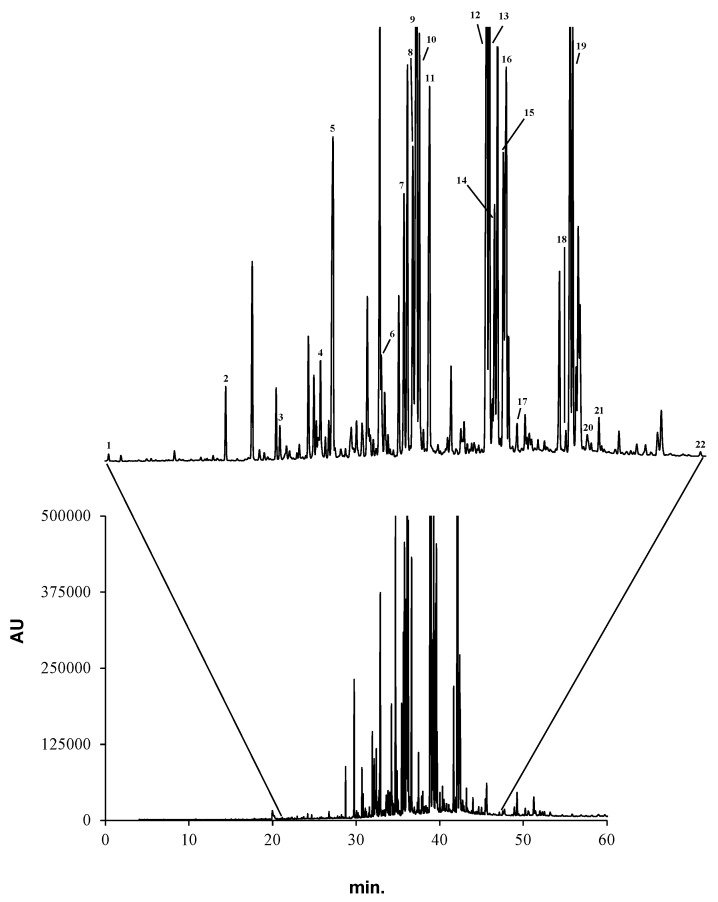
GC-MS profile of the methanol extract from the digestive gland of *A. depilans*. Peaks are identified in Table 1.

The fatty acids profile of *A. depilans* digestive gland was established by GC-MS, after saponification and derivatization to their respective methyl esters. Twenty-two fatty acids were identified, being distributed by saturated fatty acids (SFA) (dodecanoic, tetradecanoic, pentadecanoic, hexadecanoic, heptadecanoic, octadecanoic, eicosanoic, docosanoic and tetracosanoic), monounsaturated fatty acids (MUFA) (*cis*-9-hexadecenoic, *cis*- and *trans*-9-octadecenoic and *cis*-13-docosenoic) and polyunsaturated fatty acids (PUFA) (*cis*-6,9,12-octadecatrienoic, *cis*-9,12-octadecadienoic, *cis*-5,8,11,14-eicosatetraenoic, *cis*-5,8,11,14,17-eicosapentaenoic, *cis*-8,11,14-eicosatrienoic, *cis*-11,14-eicosadienoic, *cis*-9,12,15-octadecatrienoic, *cis*-4,7,10,13,16,19-docosahexaenoic and *cis*-7,10,13,16-docosatetraenoic) (Figure 2 and Table 1). To the best of our knowledge, all these compounds are described for the first time in this species.

**Table 1:** Fatty acids composition of the methanol extract from the digestive gland of *A. depilans* ^a^.

Peak	Fatty Acids	mg/kg (Dry Basis)
1	Dodecanoic	7.67 (0.26)
2	Tetradecanoic	55.60 (0.64)
3	Pentadecanoic	26.08 (1.40)
4	*cis*-9-Hexadecenoic	46.71 (1.27)
5	Hexadecanoic	525.48 (4.54)
6	Heptadecanoic	16.24 (0.55)
7	*cis*-6,9,12-Octadecatrienoic	109.88 (0.76)
8	*cis*-9,12-Octadecadienoic	132.65 (0.75)
9	*cis*-9-Octadecenoic	214.90 (4.50)
10	*trans*-9-Octadecenoic	171.13 (2.54)
11	Octadecanoic	355.70 (2.57)
12	*cis*-5,8,11,14-Eicosatetraenoic	367.30 (5.26)
13	*cis*-5,8,11,14,17-Eicosapentaenoic	270.35 (1.35)
14	*cis*-8,11,14-Eicosatrienoic	200.66 (3.36)
15	*cis*-11,14-Eicosadienoic	91.58 (0.70)
16	*cis*-9,12,15-Octadecatrienoic	115.49 (1.56)
17	Eicosanoic	21.88 (0.95)
18	*cis*-4,7,10,13,16,19-Docosahexaenoic	7.43 (0.07)
19	*cis*-7,10,13,16-Docosatetraenoic	355.99 (3.08)
20	*cis*-13-Docosenoic	14.18 (0.40)
21	Docosanoic	21.42 (0.45)
22	Tetracosanoic	3.33 (0.10)
**Total**		3131.65
**SFA**		1033.40
**MUFA**		446.92
**PUFA**		1651.33
**ω3**		393.27
**ω6**		1258.06

^a^ Results are expressed as mean (standard deviation) of three determinations. SFA, saturated fatty acids; MUFA, monounsaturated fatty acids; PUFA, polyunsaturated fatty acids.

The methanol extract from the digestive gland of *A. depilans* was essentially constituted by PUFA (*ca.* 52.7% of total fatty acids), followed by SFA (*ca.* 33% of total fatty acids) (Table 1), unlike another sea hare, *Aplysia fasciata* Poiret, which is essentially composed of SFA [20]. Contrarily, *Aplysia punctata* Cuvier was mainly composed by unsaturated fatty acids, which is in agreement with our results [20]. Concerning PUFA, the digestive gland of *A. depilans* essentially contains ω6 fatty acids (*ca.* 76% of PUFA total contents), *cis*-5,8,11,14-eicosatetraenoic acid being the major one (Table 1). Regarding SFA, hexadecanoic acid was clearly the most abundant one (*ca.* 51% of SFA total contents). This saturated fatty acid was already reported to be the main compound in *A. fasciata* and *A. punctata* [20].

Generally, many marine organisms are regarded as excellent sources of fatty acids. A previous study performed by Pereira *et al*. [21] reported that the echinoderms *Holothuria forskali* Chiaje and *Paracentrotus lividus* Lamarck are essentially composed of unsaturated fatty acids, ω6 fatty acids predominating, as it happens with our sample.

Amongst the fatty acids reported in the digestive gland of *A. depilans*, the presence of long-chain PUFA is highlighted, particularly of those with three or more double bonds, which are a class of lipids characteristic of marine organism with important bioactivities [22]. In addition, taking into account that vegetable oils do not contain these fatty acids and that the conversion of C18 PUFA to eicosapentaenoic acid (EPA) and docosahexaenoic acid (DHA) in the human body is inefficient [23], this marine organism could have a positive impact on human health.

#### 2.1.2. Carotenoids

**Figure 3:**
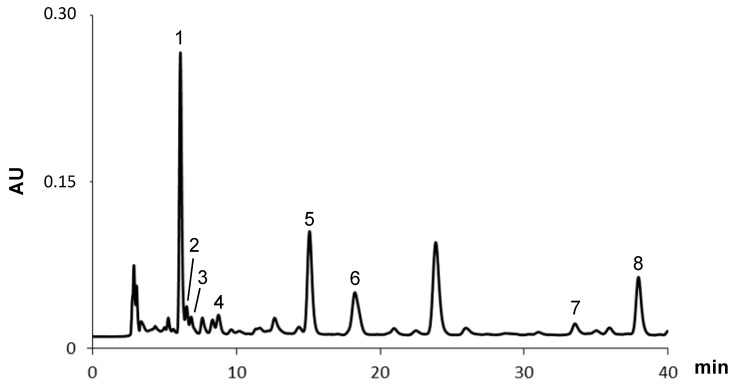
HPLC-DAD carotenoids profile of the methanol extract from the digestive gland of *A. depilans*. Detection at 450 nm. Peaks are identified in Table 2.

Eight carotenoids, namely fucoxanthin and two isomers, neoxanthin, lutein, zeaxanthin, α- and β-carotene were found in the methanol extract from the digestive gland of *A. depilans* (Figure 3 and Table 2), unlike what happened with *A. punctata*, for which no carotenoid was detected [20]. As far as we know, with the exception of lutein, the other metabolites are being described for the first time in this species. Taking into account that animals do not synthesize carotenoids, the presence of these compounds in the organism is mainly due to their direct accumulation from the diet [24]. The presence of these natural pigments in the digestive gland of this marine organism is not surprising, as it is well known that sea hares are herbivorous and feed on *Phaeophyceae* and *Rhodophyceae* (brown and red algae, respectively), being widely recognized that brown algae essentially produce zeaxanthin, β-carotene, fucoxanthin and violaxanthin, while red algae produce α- and β-carotene, zeaxanthin and lutein [24,25,26]. In fact, *A. depilans* digestive gland is characterized by the presence of high amounts of xanthophylls, namely zeaxanthin, which corresponds to 55% of the total carotenoids (Table 2).

**Table 2:** Carotenoids composition of the methanol extract from the digestive gland of *A. depilans* ^a^.

Peak	Carotenoids	μg/g (Dry Basis)
1	Fucoxanthin	215.80 (0.58)
2	Fucoxanthin *cis* isomer 1	24.50 (0.11)
3	Fucoxanthin *cis* isomer 2	15.60 (0.20)
4	Neoxanthin	12.80 (0.03)
5	Lutein	33.80 (0.04)
6	Zeaxanthin	403.40 (0.50)
7	α-Carotene	5.70 (0.01)
8	β-Carotene	18.90 (0.26)
	**Total**	**730.50**

^a^ Results are expressed as mean (standard deviation) of three determinations.

Marine organisms are known to be a rich source of carotenoids and the presence of higher amounts of xanthophylls in these organisms is not surprising. For example, the yellowish-brown echinoderm, *Coscinasterias tenuispina* Lamarck is reported to be essentially composed of zeaxanthin, similar to our marine organism [27]. Contrarily, in the brown-violet echinoderm *Holothuria tubulosa* Gmelin, and in the sea hare *A. fasciata*, isozeaxanthin is the predominant compound [27]. The green crab *Carcinus maenas* L*.* is also essentially constituted by xanthophylls, astaxanthin diester and astaxanthin monoester being the main compounds [28]. Regarding sponges, in *Acanthella acuta* Schmidt (red sponge), astaxanthin is the dominant carotenoid, while in *Tethya auratium* Pallas (lemon-green sponge), β-carotene is the main one [27].

### 2.2. Anti-Inflammatory Potential

Epidemiological studies provided convincing evidence that natural products from several sources can be interesting for the development of modern therapeutic anti-inflammatory drugs [29]. In this sense, the anti-inflammatory potential of the methanol extract from *A. depilans* digestive gland was approached on macrophage cell line RAW 264.7. Macrophages activity can be induced by certain triggers. One important exogenous trigger is LPS, a component of the cell wall of Gram-negative bacteria, which can directly activate monocytes and macrophages, leading them to produce a variety of inflammatory mediators and reactive oxygen and nitrogen intermediates [30]. First, and in order to check the potential cytotoxicity of the methanol extract, its effect on cell viability and on membrane integrity was evaluated. Thereafter, the anti-inflammatory potential was screened by assessing the extract’s capacity to reduce the levels of NO and L-citrulline present in the supernatant of the cell culture.

#### 2.2.1. Cell Viability and Membrane Integrity

**Figure 4:**
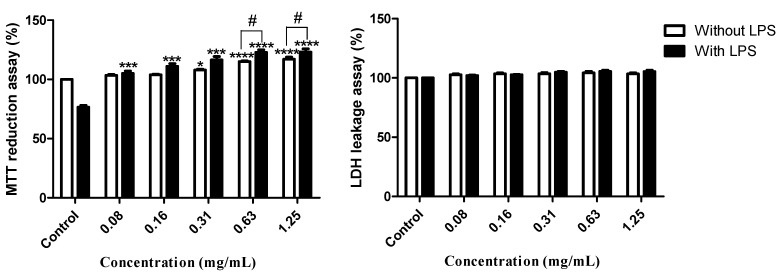
Viability of RAW 264.7 cells pre-treated for 1 h with methanol extract, followed by 18 h co-treatment with lipopolysaccharide (LPS) or LPS vehicle. Results are presented as mean ± standard error of the mean of five independent experiments, performed in duplicate. * *p* < 0.05, *** *p* < 0.01, **** *p* < 0.0001 compared to the respective control (with or without LPS); # *p* < 0.05 LPS exposed cells compared with cells exposed only to the respective extract concentration.

The effects of the methanol extract from the digestive gland of *A. depilans* on cell viability and on membrane integrity were evaluated by the MTT reduction and LDH leakage assays, respectively. As can be seen in Figure 4, the digestive gland from *A. depilans* did not reveal cytotoxicity under the tested concentrations. It was verified that exposure to LPS (1 µg/mL) for 18 h caused a decrease in cell viability to 76.6% ± 2.5% relative to unexposed cells (Figure 4). In addition, it was also observed that the digestive gland’s extract was able to restore cellular viability to control levels in cells concomitantly exposed to LPS (Figure 4).

A study performed by Pereira *et al*. [31] with RAW 264.7 macrophages demonstrated the effect of hexadecanoic, *cis*-11-eicosenoic and *cis*-11,14-eicosadienoic acids on cell viability. According to the authors, hexadecanoic acid increased LPS-induced toxicity, while *cis*-11,14-eicosadienoic acid caused a moderate reduction in LPS-induced loss of viability. Furthermore, the authors concluded that a combination of different fatty acids might restore cell’s viability after LPS exposition [31]. In addition, some studies demonstrated that carotenoids, such as fucoxanthin and lutein, did not affect RAW 264.7 cell viability [32,33]. Thus, the capacity of *A. depilans* digestive gland to restore cell’s viability may result from a combined effect of the determined compounds (Table 1 and Table 2).

#### 2.2.2. NO and L-Citrulline Levels

**Figure 5:**
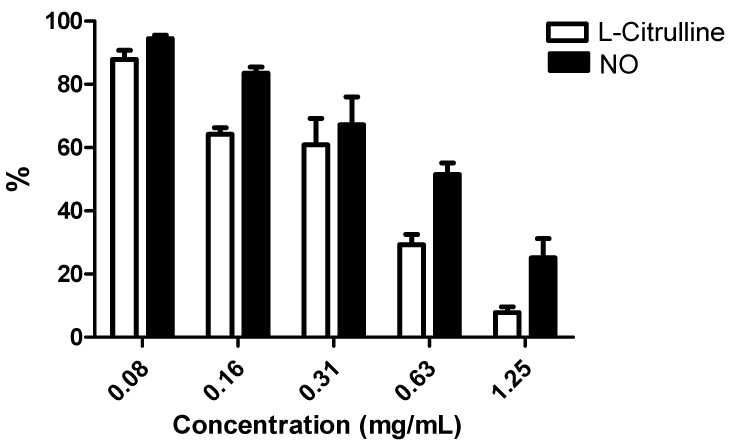
NO and L-citrulline levels on RAW 264.7 cells pre-treated for 1 h with methanol extract, followed by 18 h co-treatment with 1 µg/mL LPS. Results are presented as mean ± standard error of the mean of five independent experiments, performed in duplicate.

It is well known that macrophages exposed to LPS increase the production of NO by induction of inducible nitric oxide synthase (iNOS). In the assay without LPS, NO and L-citrulline levels in control cells and cells exposed to digestive gland’s extract were very low and could not be accurately quantified (data not shown). *A. depilans* digestive gland was able to decrease both NO and L-citrulline levels in a dose-dependent way in LPS-exposed cells (Figure 5), with IC_50_ values of 0.663 mg/mL for NO and 0.420 mg/mL for L-citrulline, which suggests that the anti-inflammatory capacity of this matrix may be due to the inhibition of iNOS activity and/or expression.

The anti-inflammatory activity of *A. depilans* digestive gland may be partially correlated with the metabolites identified in this study, mainly with the existence of unsaturated fatty acids and carotenoids. Several studies have been performed in order to evaluate the role of fatty acids in inflammation. In a general way, ω3 and ω6 PUFAs are considered to be the most powerful intracellular and intercellular mediators precursors for the synthesis of eicosanoids, which are essential for the regulation of inflammation [34,35]. It is well known that *cis*-5,8,11,14-eicosatetraenoic (arachidonic acid, ω6 PUFA) can be considered a pro-inflammatory fatty acid, once it is a precursor of the synthesis of eicosanoids, increasing the inflammatory response [30]. On the other hand, long-chain ω3 PUFAs, such as *cis*-5,8,11,14,17-eicosapentaenoic (EPA) and *cis*-4,7,10,13,16,19-docosahexaenoic (DHA) acids, are potentially interesting anti-inflammatory agents, by decreasing the production of inflammatory mediators (eicosanoids, cytokines and NO) and the expression of adhesion molecules [30,36]. EPA, DHA and *cis*-9,12,15-octadecatrienoic (α-linolenic) acids are also known to inhibit iNOS expression [34,37,38]. In fact, as can be observed in Table 1, the digestive gland from *A. depilans* contains high amounts of pro-inflammatory arachidonic acid (367.30 mg/kg of dry weight), which can be balanced by the high amounts of EPA, DHA and α-linolenic (393.27 mg/kg of dry weight). In addition, hexadecanoic acid is known for its capacity to induce the expression of pro-inflammatory markers as a consequence of the activation of Toll-like receptor 4 (TLR4) [39]. Therefore, some fatty acids may partially contribute to the anti-inflammatory activity.

Carotenoids have also been shown to possess anti-inflammatory capacity. In fact, zeaxanthin, the major xanthophyll found in the digestive gland of *A. depilans* (403.40 and µg/g, dry weight, Table 2), is known to inhibit iNOS expression [40]. In addition, it has been previously demonstrated by Heo and collaborators [32] that fucoxanthin, the second major xanthophyll in the digestive gland of *A. depilans* (255.90 and µg/g, dry weight, Table 2), has significant anti-inflammatory activity. According to those authors, this activity may be correlated with its capacity to inhibit NO production, as well as with its ability to inhibit the expression of iNOS, cyclooxygenase 2 and pro-inflammatory cytokines [32]. Lutein and β-carotene are also known to inhibit NO production, by decreasing iNOS expression [33,41]. Therefore, the carotenoids identified in this work should also contribute to the overall effects.

## 3. Experimental Section

### 3.1. Standards and Reagents

Authentic standards of fatty acids methyl esters for GC-MS analysis were obtained from Supelco (Bellefonte, PA, USA). Fucoxanthin, lutein, zeaxanthin and β-carotene were from Sigma (St. Louis, MO, USA). α-Carotene and neoxanthin were from CaroteNature (Lupsingen, Switzerland).

LPS from *Salmonella enterica*, sodium pyruvate, thiazolyl blue tetrazolium bromide (MTT), β-nicotinamide adenine dinucleotide reduced form (NADH), sodium nitroprusside (SNP), sulfanilamide, *N*-(1-naphthyl) ethylene diamine, isooctane, methanol and dimethyl sulfoxide (DMSO), BF_3_ and KOH, were from Sigma-Aldrich (St. Louis, MO, USA). Dulbecco’s Modified Eagle Medium (DMEM), Dulbecco’s phosphate buffered saline (DPBS), heat inactivated fetal bovine serum (FBS) and Pen Strep solution (Penicillin 5000 units/mL and Streptomycin 5000 mg/mL) were purchased from Gibco, Invitrogen™ (Grand Island, NY, USA).

### 3.2. Sampling

Specimens of *A. depilans* were randomly collected during the low tide on the seashore of Oporto city. The animals were placed in seawater and transported to the laboratory at room temperature. Shortly thereafter, the organisms were cleaned, washed with seawater and dissected. Samples were kept at −20 °C, prior to freeze-drying in a Virtis SP Scientific Sentry 2.0 apparatus (Gardiner, NY, USA). The dried samples were ground (particle size < 910 µm) before use.

### 3.3. Extraction

Powdered digestive glands of *A. depilans* (1000 ± 10.0 mg) were mixed with methanol (20 mL) under magnetic stirring (300 rpm), for 20 min, at 40 °C. The extract was filtered through a 0.45 μm membrane (Millipore, Billerica, MA, USA).

### 3.4. Fatty Acids Determination

#### 3.4.1. Derivatization Process

Two and a half milliliters of extract were concentrated to dryness under reduced pressure (40 °C). The residue was then hydrolyzed with 1 mL of KOH methanol solution (11 g/L), at 90 °C, for 10 min. The free fatty acids originally present and those resulting from alkaline hydrolysis were derivatized to their methyl esters (FAME) with 1 mL of BF_3_ methanol solution (10%), at 90 °C, for 10 min. FAME were purified with 2 × 6 mL of isooctane and anhydrous sodium sulfate was added to assure the total absence of water. The resulting extract was evaporated to dryness under a stream of nitrogen and dissolved in 100 mL of isooctane. The extract was analyzed in triplicate.

#### 3.4.2. GC-MS Analysis

GC-MS analysis was performed following a previously established method [42], with some modifications. Derivatized extracts (2 µL) were analyzed using a Varian CP-3800 gas chromatographer (USA) equipped with a Varian Saturn 4000 mass selective detector (USA) and a Saturn GC/MS workstation software version 6.8. A VF-5 ms (30 m × 0.25 mm × 0.25 µm) column (Varian) was maintained at 40 °C for 1 min, then the temperature increased 5 °C/min to 250 °C, 3 °C/min to 300 °C and held for 15 min. All mass spectra were acquired in electron impact (EI) mode. Ionization was maintained off during the first 4 min, to avoid solvent overloading. The Ion Trap detector was set as follows: transfer line, manifold and trap temperatures were, respectively, 280, 50 and 180 °C. The mass ranged from 50 to 600 *m*/*z*, with a scan rate of 6 scan/s. The emission current was 50 µA and the electron multiplier was set in relative mode to auto tune procedure. The maximum ionization time was 25,000 µs, with an ionization storage level of 35 *m*/*z*. The analysis was performed in Full Scan mode. The identification of compounds was achieved by comparison of their retention indices and mass spectra with those from pure standards, injected under the same conditions, and from NIST 05 MS Library Database. The amount of FAME present in the samples was achieved from the calibration curve of the respective standard prepared in isooctane.

### 3.5. Carotenoids Determination

#### HPLC-DAD Analysis

Carotenoids were separated on a C30 YMC column (5 µm, 250 × 4.6 mm i.d.; YMC, Kyoto, Japan), at room temperature, according to a previously described procedure [43], with some modifications. The mobile phase consisted of two solvents: methanol (A) and *tert*-butyl methyl ether (B). Elution started with 95% A and a gradient was used to obtain 70% A at 30 min, followed by 50% A at 50 min. The injection volume was 20 µL and the flow rate 0.9 mL/min. Elution was monitored at 450 nm. Carotenoids quantification was achieved by the absorbance recorded in the chromatograms relative to reference standards analyzed under the same sample conditions. The isomers of fucoxanthin were quantified as fucoxanthin.

### 3.6. Anti-Inflammatory Potential

#### 3.6.1. Cell Culture

RAW 264.7 macrophage cells were from the American Type Culture Collection (LGC Standards S.L.U., Barcelona, Spain) and were maintained in DMEM supplemented with 10% FBS and 2% penicillin/streptomycin at 37 °C, in a humidified atmosphere of 5% CO_2_, as previously reported [44]. Cells were seeded in 48-well plates (90,000 cells/well) and cultured until 80%–90% confluence. Cells were pre-treated with different concentrations of extract from *A. depilans* digestive glands dissolved in medium with 0.5% DMSO or vehicle, for 1 h, and then treated with 1 µg/mL LPS (or vehicle), for 18 h at 37 °C, in a humidified atmosphere of 5% CO_2_.

#### 3.6.2. Cell Viability

Cell viability was evaluated by the MTT reduction assay [44]. In metabolically active cells, MTT is converted to formazan and the extent of formazan production was quantified by measuring the absorbance at 510 nm in a microplate reader (Multiskan ASCENT). The results of cell viability correspond to the mean of five independent experiments performed in duplicate, and are expressed as percentage of the untreated control cells.

#### 3.6.3. Membrane Integrity

Lactate dehydrogenase (LDH) released into the culture media was determined by measuring the decrease of NADH at 340 nm during the conversion of pyruvate to lactate [44]. Results are expressed as percentage of the respective control (with or without LPS). Five independent experiments were performed in duplicate.

#### 3.6.4. NO Determination

The nitrite resulting from the conversion of NO in the culture medium was measured by the Griess reaction [44]. Five independent experiments were performed in duplicate. Control values were obtained in the absence of digestive glands extract.

#### 3.6.5. L-Citrulline Determination

The quantification of L-citrulline was based on a method previously described [45], with some modifications. Briefly, after 18 h incubation, 250 µL of culture medium were mixed with 100 µL of a reaction mixture containing diacetylmonoxime, antipyrine E and H_2_SO_4_. After incubation at 96 °C for 25 min in a block heater (Stuart SBH 200D/3, Staffordshire ST 15 0SA, UK), the solution was cooled down to room temperature and the absorption was read at 405 nm. Five independent experiments were performed in duplicate.

### 3.7. Statistical Analysis

Statistical analysis was performed using GraphPad Prism 6 Software (San Diego, CA, USA). One-way ANOVA and Bonferroni’s test, as *post-hoc* test, were used to determine the statistical significance in comparison to control. Two-way ANOVA and Sidak’s multiple comparison tests were used to determine the differences between extract and LPS in cell viability and between L-citrulline and NO release in LPS-stimulated macrophages. In all cases, *p* values lower than 0.05 were considered statistically significant.

## 4. Conclusions

Studies exploiting the chemical composition of *A. depilans* are scarce. Thirty compounds were determined for the first time in its digestive gland. In a general way, the anti-inflammatory capacity displayed by this material seemed to be correlated with the determined metabolites. Nevertheless, the presence of other non-determined compounds, which can also contribute for the observed activity, cannot be ignored. Overall, the results indicate that the digestive gland from *A. depilans* may be an interesting source of nutraceuticals, namely carotenoids and long-chain PUFA, providing beneficial anti-inflammatory effects. Further studies involving other markers for inflammation would be interesting to explore the mechanisms behind the anti-inflammatory activity of this species.
